# Adaptive Defense Mechanism During Flowering Period of *Rhododendron decorum* Revealed by Comparative Transcriptomic Analysis

**DOI:** 10.3390/plants14040559

**Published:** 2025-02-12

**Authors:** Weiwei Liu, Chenghua Yu, Kaiye Yang, Ling Wang, Lianming Gao, Xinchun Mo

**Affiliations:** 1 Lijiang Forest Biodiversity National Observation and Research Station, Kunming Institute of Botany, Chinese Academy of Sciences, Lijiang 674100, China; liuweiwei@mail.kib.ac.cn (W.L.); yangkaiye@mail.kib.ac.cn (K.Y.); 2 School of Applied Technology, Lijiang Normal University, Lijiang 674199, China; 18468182962@163.com (C.Y.); wangling1014@163.com (L.W.); 3 State Key Laboratory of Plant Diversity and Specialty Crops, Kunming Institute of Botany, Chinese Academy of Sciences, Kunming 650201, China; 4 College of Forestry, Shandong Agricultural University, Tai’an 271018, China

**Keywords:** comparative transcriptomic analysis, adaptive defense mechanism, functional disparities, flowering period, *Rhododendron decorum*

## Abstract

*Rhododendron decorum*, a widely distributed *Rhododendron* species in southwestern China, is recognized not only for its significant ornamental value but also as a culinary resource for local tribes. However, the defense mechanisms underlying the ecological adaptations of *R. decorum* remain to be elucidated. In this study, we conducted comparative transcriptomic analyses of various organs (corolla, androecium/gynoecium and leaves) of *R. decorum* collected from two distinct two regions. Approximately 186.98 Gb of clean data were generated from three organs of *R. decorum* across these regions. Through de novo assembly, a total of 92,025 unigenes were obtained and nearly half of them (43,515 unigenes) were successfully annotated. Enrichment analysis of differentially expressed genes (DEGs) within three comparative groups of different organs (HQI/LFI, HQO/LFO and HQL/LFL, respectively) revealed that the distribution of *R. decorum* in the Heqing region exhibited an increased requirement for plant immunity, including resistance to diseases, insects, and herbivores across various plant organs. Conversely, *R. decorum* in the Lijiang region showed a greater reliance on environmental factors, such as cold tolerance, aromatic compounds production, and the attraction of pollinating insects. Notably, the validation of 21 pivotal genes identified from significantly regulated enrichment pathways across different organs showed functional consistency in the KEGG enrichment analysis among different organs in these two regions. The functional disparities observed in the transcriptome of *R. decorum* across distinct regions provide valuable insight into the understanding of its adaptive defense mechanism.

## 1. Introduction

*Rhododendron* L. is the largest genus of woody plants in the northern hemisphere and the largest genus of spermatophytes in China, comprising over 600 species predominantly found in the Hengduan Mountains and the Himalayas, which are recognized as significant centers for species distribution and diversification [1]. Members of this genus possess considerable horticultural value due to their vibrant and diverse coloration. Moreover, certain species of *Rhododendron* flowers have been extensively utilized in traditional food and/or medicine practices in Yunnan Province, China [2]. These flowers contain a diverse array of chemical compounds, which exhibit various medicinal properties, including cough suppression, enhancement of expectoration, alleviation of asthma symptoms, the reduction of blood pressure and cholesterol levels, promotion of diuresis, and antibacterial effects [3,4,5,6,7,8]. *Rhododendron decorum*, in particular, is prevalent in several ethnic minority regions of Yunnan Province, where it is utilized by local communities for medicinal purposes and as a functional food. However, significant variations in its use and application can be observed among different ethnic groups [9]. For instance, the corolla, with the removal of the androecium and gynoecium, is consumed as a culinary delicacy in regions inhabited by the Bai ethnic group, where the consumption of flowers holds deep-rooted cultural significance [2].

In the realm of modern plant transcriptomic analysis, it is suggested that the same species may exhibit distinct transcriptional processes and mediate the production of diverse metabolites across different regions [10]. Such variations may arise from environmental factors, including temperature, moisture, light exposure, soil composition, or specific microbial influences [11]. Transcriptional regulation is a well-studied mechanism in organisms and has been extensively investigated to assess various physiological characteristics of plants [12]. Comparative transcriptomic approaches can be effectively utilized to evaluate ecological adaptability. Through comparative transcriptomic analyses of species within the genus *Rhododendron*, differential gene expression patterns have been observed related to the accumulation of medicinal compounds, elucidation of mechanisms responsible for variations in flower color, comparison of stress resistance levels, and identification of alterations in transcription factors associated with carbohydrate metabolism relevant to functional foods [13,14,15,16,17].

Despite numerous transcriptomics analyses reporting response to environmental stress and changes in flower color, the gene expression patterns associated with the differential accumulation of metabolites during the flowering period remain largely unexplored. We evaluated the metabolites of *R. decorum* from two regions and observed differential accumulation trends in our previous study, despite the similarity of environmental factors in these areas over a short time frame [13]. Therefore, in this study, we aim to elucidate the variations in transcript levels of *R. decorum* from the same regions by differential transcriptomic analyses of its diverse floral organs. We conducted a comparative transcriptomic analysis of three organs (corolla, androecium/gynoecium, and leaf) in *R. decorum* from Heqing and Yulong counties to explore the differential expression patterns, which may provide insights into the adaptive defense mechanisms in response to ecological environments during the flowering period of *R. decorum*.

## 2. Results

### 2.1. Sequencing and Transcription Data Statistics

Six cDNA libraries were constructed using the total RNA derived from the leaves and flowers (including corolla and androecium/gynoecium) of *R. decorum* collected from Heqing County (HQ), Dali Bai Autonomous Prefecture and Yulong Naxi Autonomous County (LF) region, respectively. These two regions are 54.85 km apart. To minimize habitat heterogeneity, matured *R. decorum* plants were collected from locations with similar climatic conditions, comparable altitudes, identical vegetation types and consistent slope orientations and positions. Approximately 186.98 Gb raw data were generated. The quality assessment of the raw reads indicated that 92.88% of the reads exhibited a base quality surpassing Q30 and were subsequently trimmed prior to assembly (Table S1). After the removal of adapter, low quality reads and short reads (<50 bp), over 21 million high-quality reads were obtained from the corolla, androecium/gynoecium, and leaf libraries, respectively. Then, the clean data were used to conduct de novo assembly, and a total of 92,025 unigenes were generated, with an average length of 880 bp. After the redundant clusters were eliminated, the resulting unigenes had a N50 length of 1604 bp and a GC content of 40.67% (Table S2). Most of the assembled unigenes were found to distribute between 200 bp and 3000 bp (Figure S1A). Among them, nearly half of them (47.84%, 44,025 unigenes) were longer than 500 bp in length, where over a quarter of unigenes were (25.71%, 23,664 unigenes) longer than 1000 bp. Additionally, a total of 60,781 coding sequences (CDSs) were predicted, including 25,020 (41.16%) complete CDSs. Among them, 18,127 (29.84%) CDSs were longer than 500 bp in length (Table S3, Figure S1B). The sequences were highly qualified and favored subsequent functional annotations.

### 2.2. Gene Functional Annotation and Expression Analysis

To address the functional annotation of the assembled unigenes, the identified unigenes were blasted against the eight public databases for sequence similarity. Results showed that nearly half of unigenes (43,515 unigenes, 47.29%) had significant matches in these databases, where others were uninformative. Among these annotations, a maximum annotation (44.09%) was observed against the NR database while the COG had the least number of annotated unigenes (9.09%). Also, over 20% of unigenes acquired significant hits with six databases, such as eggNOG (32.76%), GO (29.58%), Pfam (27.19%), Swissprot (25.56%), KEGG (25.39%), and KOG (21.64%), respectively (Table 1).

**Figure 1:**
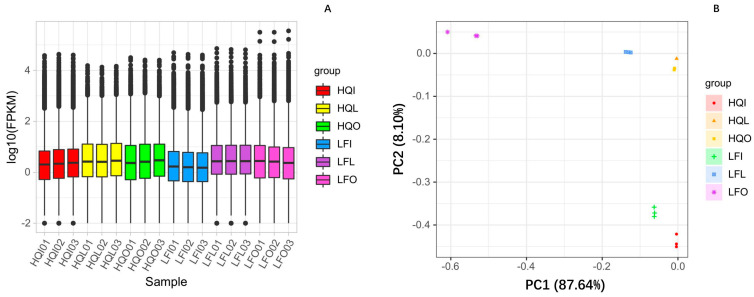
FPKM values estimation and PCA of different organs of *Rhododendron decorum* from HQ and LF regions. (**A**) The FPKM box plots of gene expression levels. (**B**) The PCA plot of gene expression levels.

Fragments per Kilobase of exon model per Million mapped fragments (FPKM) values were estimated and principal component analysis (PCA) was performed. Results showed that the androecium/gynoecium group (HQI/LFI) exhibited slightly lower gene expression levels compared to the leaves (HQL/LFL) group and corolla group (HQO/LFO) (Figure 1A; Table S4). In terms of gene expression levels, PCA results explained 87.64% of the observed inter-group differences within the first principal component, while the second principal component accounted for 8.10% of these differences, indicating the high explanatory power of the model. Among different regions, there was a similar distribution pattern in gene expression levels between leaves (HQL/LFL) group and androecium/gynoecium group (HQI/LFI), whereas the corolla group (HQO/LFO) exhibited significant variations in gene expression level distribution patterns across regions (Figure 1B). Subsequent analyses mainly focus on differential gene expression and enrichment analyses among different regions within the same plant organ.

### 2.3. The Differences in Transcriptome Gene Expression

By comparing the overlap of differentially expressed genes (DEGs) between groups, 4712 unique DEGs were observed in the HQL/LFL group, which possessed the highest number of unique DEGs, where the HQI/LFI and HQO/LFO groups had 2216 and 2566 unique DEGs, respectively. The HQL/LFL and HQI/LFI groups exhibited a shared repertoire of 1763 DEGs, while the HQL/LFL and HQO/LFO groups also displayed an overlapping set of 1763 DEGs. Moreover, the HQI/LFI and HQO/LFO groups demonstrated a common subset of 1378 DEGs. Notably, the three groups collectively presented a total of 792 commonly regulated genes (Figure S2A).

In the pairwise comparisons of DEGs within three groups (HQI/LFI, HQL/LFL, HQO/LFO), the HQI group showed a total of 4565 DEGs compared to the LFI group, with 2571 upregulated genes and 1994 downregulated genes. Moreover, the HQL group exhibited a total of 7446 DEGs in comparison to the LFL group, consisting of 4515 upregulated genes and 2931 downregulated genes. Additionally, the HQO group displayed a total of 4915 DEGs when compared to the LFO group, with 1927 upregulated genes and 2988 downregulated genes (Figure S2B).

**Figure 2:**
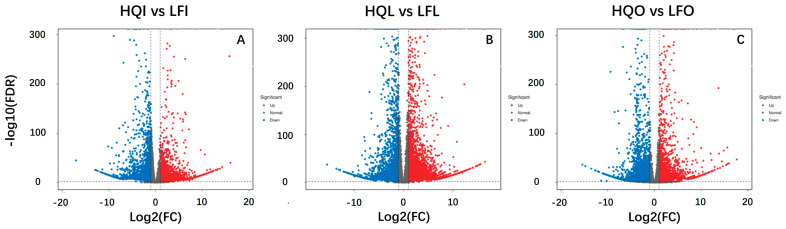
The volcano plot of differential expression genes in different group comparison. (**A**) Differential expression of genes in HQI/LFI group. (**B**) Differential expression of genes in HQL/LFL group. (**C**) Differential expression of genes in HQO/LFO group.

The volcano plot showed a more intuitive visualization of the distribution of differentially expressed genes between groups, as well as the magnitude and statistical significance of gene expression alterations (Figure 2). The HQI/LFI and HQL/LFL groups showed an abundance of upregulated genes, while the HQO/LFO group exhibited a prevalence of downregulated genes. Furthermore, it is worth noting that the HQL/LFL group exhibited the highest number of differentially expressed genes.

### 2.4. Enrichment Analysis of Differentially Expressed Transcriptome Genes

The KEGG enrichment analysis of differentially expressed genes revealed that the differential expression gene KEGG classifications remain consistent across various groups (HQI/LFI, HQL/LFL, HQO/LFO) at the first level of KEGG classification, encompassing cellular processes, environmental information processing, genetic information processing, metabolism, and organismal systems, where similar patterns were also observed at the second level of KEGG classification (Figure S3). Interestingly, the floral organs (corolla and androecium/gynoecium) in the HQ group exhibited high differential expression in phagosome within cellular processes (Figure S3A,C). Additionally, all groups (HQI/LFI, HQL/LFL, HQO/LFO) exhibited a higher proportion of differentially expressed genes in the plant–pathogen interaction pathway (Figure S3).

**Figure 3:**
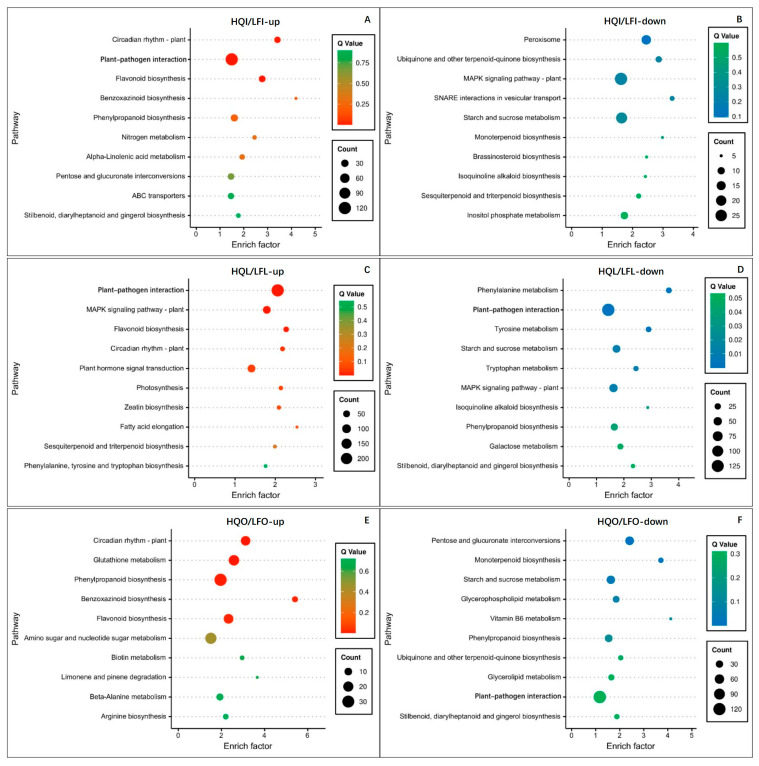
KEGG enrichment analysis of differentially expressed genes in differential comparison of different groups. (**A**) The top 10 enrichment KEGG pathways of upregulated differentially expressed genes in HQI/LFI group. (**B**) The top 10 enrichment KEGG pathways of downregulated differentially expressed genes in HQI/LFI group. (**C**) The top 10 enrichment KEGG pathways of upregulated differentially expressed genes in HQL/LFL group. (**D**) The top 10 enrichment KEGG pathways of downregulated differentially expressed genes in HQL/LFL group. (**E**) The top 10 enrichment KEGG pathways of upregulated differentially expressed genes in HQO/LFO group. (**F**) The top 10 enrichment KEGG pathways of downregulated differentially expressed genes in HQO/LFO group. The y-axis denotes the pathway name, while the x-axis indicates the Enrichment Factor—a ratio of genes annotated to a specific pathway in differentially expressed genes versus all genes. The color of each point reflects its q-value, with smaller values indicating more reliable enrichment significance for differentially expressed genes within that pathway. Additionally, larger points represent pathways with more enriched genes. The diagram illustrates the 10 pathways with the lowest q-values.

In the HQI and LFI groups, the upregulated DEGs were significantly enriched in three KEGG pathways: circadian rhythm—plant, plant–pathogen interaction, and flavonoid biosynthesis (Figure 3A, Table S5). Specifically, the plant–pathogen interaction pathway exhibited a high GeneRatio of functionally differentially expressed genes, with 18.72% (Table S5), indicating a robust immune response against plant pathogens in the HQI group compared to the LFI group with regard to androecium/gynoecium. The collective upregulation of three distinct gene enrichment pathways accounted for 25% of all functionally differentially expressed genes (Table S5). Conversely, the enrichment analysis of differentially downregulated gene pathways within the HQI and LFI groups revealed a significant enrichment observed in four KEGG pathways: peroxisome, ubiquinone and other terpenoid-quinone biosynthesis, MAPK signaling pathway—plant, and SNARE interactions in vesicular transport (Figure 3B, Table S5, *p*-value < 0.01). However, upon calculation using the Q-value (>0.05), the results indicated false positives in the enrichment analysis, necessitating their exclusion due to elevated Q-values. Consequently, no statistically significant differences were observed in the various pathways between the LFI and the HQI group concerning differentially downregulated genes.

DEGs were clearly displayed in the HQL/LFL group through KEGG enrichment analysis; they showed quite different enrichment patterns within these two regions, suggesting that the leaves of *R. decorum* in the HQ and LF regions might have inverse effects on response to the environment of a habitat. Results showed that the upregulated genes in the HQL/LFL group exhibited significant enrichment in five pathways: plant–pathogen interaction, MAPK signaling pathway—plant, flavonoid biosynthesis, circadian rhythm—plant, and plant hormone signal transduction (Figure 3C, Table S5). These pathways collectively accounted for nearly half (45.86%) of all functionally annotated differentially expressed genes (Table S5). In terms of their represented functions, plant immune regulation emerges as the most pivotal one, which is also intricately linked with stress-induced cellular responses (MAPK signaling pathway—plant), synthesis of resistance compounds (flavonoid biosynthesis), flowering physiology in plants (circadian rhythm—plant), and innate immunity in plants (plant hormone signal transduction). However, the down-regulated genes in the HQL/LFL group were significantly enriched in another six pathways: phenylalanine metabolism, plant–pathogen interaction, tyrosine metabolism, starch and sucrose metabolism, tryptophan metabolism, and MAPK signaling pathway—plant (Figure 3D, Table S5). However, both the plant–pathogen interaction and MAPK signaling pathway were found to enrich many up-regulated genes in the HQL/LFL group, suggesting that the expression of DEGs was associated with plant immune response and stress-induced cellular responses in both HQL and LFL samples. Interestingly, numerous differentially expressed genes in the LFL group were found to be enriched in pathways such as phenylalanine metabolism, tyrosine metabolism, and tryptophan metabolism.

Upon comparing the enrichment of upregulated differentially expressed genes in the HQO/LFO groups across various pathways, it was evident that the primary enriched pathways encompassed the circadian rhythm—plant, glutathione metabolism, phenylpropanoid biosynthesis, benzoxazinoid biosynthesis, and flavonoid biosynthesis (Figure 3E, Table S5). The cumulative GeneRatio values of 15.94% from these pathways showed that, despite minor variations in the enrichment of functional genes between the two corolla groups, they still exhibited distinct functionalities. The pathway “Circadian rhythm—plant” reflected the flowering pattern in plants, whereas pathways such as “Glutathione metabolism”, “Phenylpropanoid biosynthesis”, “Benzoxazinoid biosynthesis”, and “Flavonoid biosynthesis” highlighted the antioxidant and disease/pest resistance properties. In the HQL/LFL group, the down-regulated genes showed significant enrichment in three pathways: “Pentose and glucuronate interconversions”, “Monoterpenoid biosynthesis”, and “Starch and sucrose metabolism” (Figure 3F, Table S5).

### 2.5. Identification and Validation of Pivotal Genes in Differentially Enriched Pathways

**Figure 4:**
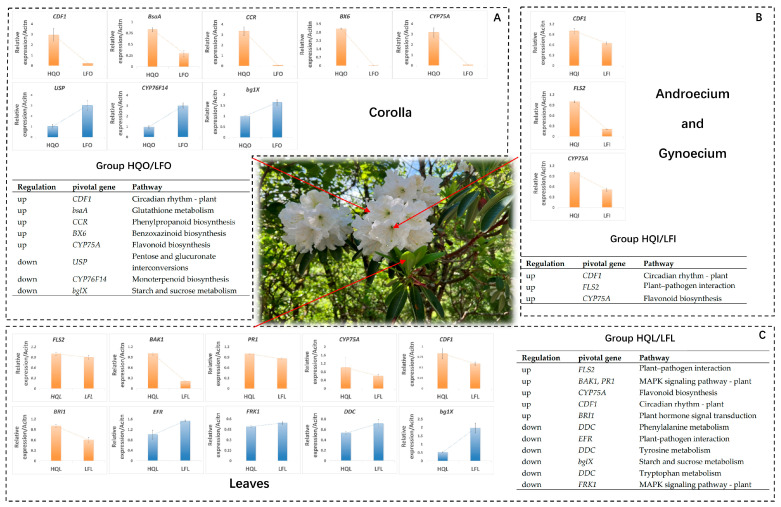
Validation results of pivotal genes selected under enriched pathways among different groups (HQI/LFI, HQL/LFL, and HQO/LFO) for *R. decorum*. (**A**) The expression patterns and gene information of identified pivotal genes in corollas groups. (**B**) The expression patterns and gene information of identified pivotal genes in androecium/gynoecium groups. (**C**) The expression patterns and gene information of identified pivotal genes in leaves groups. The validation results of different groups are represented by distinct dashed boxes. The orange bar chart illustrates the validation results of pivotal genes in the upregulated mode, while the blue bar chart represents the validation results in the downregulated mode. The table within the figure presents pivotal genes enriched in group-specific pathways.

To identify pivotal genes in the enriched pathways exhibiting differential regulatory patterns (upregulated or downregulated) among the three groups HQI/LFI, HQL/LFL, and HQO/LFO, we performed a selection based on Q-value values < 0.05 for the differentially enriched pathways to mitigate false positives. The selected enriched pathways, pivotal genes, and their primary functions are presented in Figure 4.

For the corollas, the pivotal genes identified in the enriched pathways of the circadian rhythm—plant pathway, glutathione metabolism pathway, phenylpropanoid biosynthesis pathway, benzoxazinoid biosynthesis pathway, and flavonoid biosynthesis pathway, which exhibited upregulated differential expression, were *CDF1*, *BsaA*, *CCR*, *BX6*, and *CYP75A*, respectively. In contrast, for the downregulated differentially expressed genes, the pivotal genes identified in the enriched pathways of pentose and glucuronate interconversions pathway, monoterpenoid biosynthesis pathway, and starch and sucrose metabolism pathway were *USP*, *CYP76F14*, and *bglX*, respectively (Figure 4A).

In the androecium/gynoecium group, exclusively pathways with upregulated differentially expressed genes were identified as enriched. Notably, the *CDF1*, *FLS2*, and *CYP75A* genes were identified as key regulators in the circadian rhythm—plant pathway, plant–pathogen interaction pathway, and flavonoid biosynthesis pathway, respectively (Figure 4B).

For the leaves group, the *FLS2* gene was identified as a pivotal gene for the HQL group within the shared differentially expressed gene enrichment pathway of plant–pathogen interaction, owing to distinct regulatory patterns (Figure 4C). Similarly, the *EFR* gene was also recognized as a pivotal gene for the LFL group in this pathway. Within the shared differentially expressed gene enrichment pathway of the MAPK signaling pathway—plant, the *BAK1* and *PR1* genes were identified as pivotal genes for the HQL group, based on diverse regulatory patterns. Conversely, the *FRK1* gene was identified as the pivotal gene for the LFL group in this pathway. In other upregulated differentially expressed gene enrichment pathways, the *CYP75A*, *CDF1*, and *BRI1* genes were, respectively, identified as the pivotal genes for the flavonoid biosynthesis pathway, the circadian rhythm—plant pathway, and the plant hormone signal transduction pathway, respectively. The *DDC* gene was recognized as a common pivotal gene for the phenylalanine metabolism pathway, the tyrosine metabolism pathway, and the tryptophan metabolism pathway in other downregulated differential expression enriched pathways. Finally, the *bglX* gene was identified as the key gene specifically associated with starch and sucrose metabolism.

## 3. Discussion

The medicinal potential of certain *Rhododendron* species is increasingly recognized by researchers and practitioners [4,18,19,20]. However, Yunnan Province is characterized by a high diversity of *Rhododendron* species and various ethnic groups exhibit differing attitudes towards the collection and consumption of *Rhododendron* flowers [9,19]. Through comparative transcriptomic analyses of various organs of *R. decorum* in the Bai and Naxi ethnic regions, we have identified the presence of different adaptive defense mechanisms among diverse populations of *R. decorum*. Additionally, alterations in the composition of *Rhododendron* metabolites have been documented in response to changes in external environment [21]. The application of comparative transcriptomics across multiple organs facilitates the investigation of the medicinal components of Rhododendrons, particularly in relation to plant defense mechanism.

### 3.1. The Transcriptional Characteristics of Leaves Exhibit Regional Variations

In this study, both the plant–pathogen interaction pathway and the MAPK signaling pathway in leaves exhibited a significant enrichment of differentially expressed genes in both HQL and LFL groups. The *FLS2* gene in the plant–pathogen interaction pathway was identified as the pivotal gene for the HQL group, while for the LFL group it was the *EFR* gene. The *BAK1* gene was recognized as an essential component of the MAPK signaling pathway within plants. The *FLS2* and *EFR* genes have been shown to play roles in the perception of bacterial flagellin [22,23], whereas *BAK1* is involved in the self-activation signaling pathway of plant immunity [24,25]. Moreover, the *PR1* gene was identified as a late immune response gene to plant pathogens in the HQL group [26], while *FRK1* was designated as an early immune response gene in the LFL group [27]. Notably, even within the same pathway, distinct differences in gene enrichment were observed between the HQL and LFL group. These findings suggest that the HQL group possessed a more pronounced response in terms of plant pathogen defense and signal transduction mechanisms. Results from genetic validation revealed that although both HQL and LFL groups displayed comparable expression levels of pivotal genes associated with plant immune responses, the HQL group exhibited a significantly superior performance in plant pathogen defense and signal transduction mechanisms than the LFL group. In plants, the early immune response involves the activation of immune reaction through the perception and recognition of pathogen-associated molecular patterns during the initial stages of pathogen invasion. Conversely, the late immune response includes the synthesis of antimicrobial compounds, such as flavonoids [28], which will be further investigated concerning their enrichment effect on antimicrobial compound synthesis pathways in the HQL group. From the perspective of pathway enrichment results for plant pathogen defense and signal transduction, one plausible explanation for the observed differences is that the sampling locations for the LFL group, situated at higher altitudes with lower temperatures, may reduce the impact of pathogens on *R. decorum* [29,30].

Compared to the LFL group, the HQL group exhibited distinct enrichment pathways, including flavonoid biosynthesis, circadian rhythm regulation, and plant hormone signal transduction. Three genes, namely *CYP75A*, *CDF1*, and *BRI1*, were identified as pivotal within these pathways. In the flavonoid biosynthesis pathway, the *CYP75A* gene was directly involved in the synthesis of eriodictyol, which subsequently facilitated the production of dihydroquercetin [31,32]. Dihydroquercetin not only possesses intrinsic antioxidant and anti-inflammatory properties but also aids in the biosynthesis of essential antibacterial and antioxidant compounds such as myricetin, (+)-gallocatechin, and (+)-catechin, all influenced by the *CYP75A* gene [33,34,35]. Recent studies have demonstrated that myricetin exhibited a range of beneficial physiological functions, including anti-hyperlipidemic activity and significant detoxification effects [36,37,38]. Moreover, (+)-gallocatechin exhibited significant cellular antioxidant activity and plays a crucial role in mitigating diabetes-related complications [39,40]. The widespread use of (+)-catechin can be attributed to its robust antioxidant properties, antiviral activities, and substantial pharmacological effects [41,42,43].

In the circadian rhythm—plant pathway, *CDF1* acted as a transcriptional repressor of the CONSTANS (CO) flowering gene [44]. The CO protein is a critical component among various flowering-associated genes and proteins, with its expression in many plant species being influenced by the light and dark cycle [45]. We identified the CYCLING DOF FACTOR 1 (*CDF1*) gene in HQL/LFL, which exhibited high expression levels in HQ (Figure 3C). However, the high expression of the CDF1 gene, as an expression pattern, was affected by numerous factors, and the genuine causes of its high expression and biological functions were highly challenging to determine. Further investigation is required to verify the role of the *CDF1* gene in leaf tissues of *R. decorum*. In addition, the plant–pathogen interaction pathway was found to be enriched in *R. decorum*, showing differential enrichment patterns (Figure 3). This pathway is associated with plant immunity and activates the response to environment change. Numerous studies have sought to clarify whether the rhythmic transcription of defense-related genes represents an adaptive and functional response or is simply a consequence of general genomic regulation or proximity to regulatory elements [46]. Furthermore, abiotic environmental factors such as light, diurnal cycles, and temperature may influence plant defense mechanisms against pathogen infections [47].

Within the plant hormone signal transduction pathway, the *BRI1* gene modulated the receptors for brassinosteroids, which are essential not only for regulating plant growth but also for enhancing plant defense mechanisms against environmental stress [48]. The upregulation of the *BRI1* gene in the HQL group may suggest enhanced disease resistance in *Rhododendron*, as evidenced by the significant enrichment of differentially expressed genes in the flavonoid biosynthesis pathway [49]. Furthermore, this upregulation pattern of the *BRI1* gene may also indicate a potential negative regulation of Rhododendrons’ response to cold stress in the plant hormone signal transduction pathway [50].

Pathway enrichment analysis of differentially expressed genes in the HQL/LFL group indicated both partially overlapping enriched pathways and distinct pathways with varied functions. Compared to the HQL group, the LFL group demonstrated a significant enrichment of differentially expressed genes in aromatic amino acid metabolic pathways. The critical role of the *DDC* gene in regulating these metabolic pathways has been identified [51]. These processes and products were likely involved in plant–microbe interactions and plant defense mechanisms, which had been documented in *Rhododendron* [52,53]. In comparison to the HQL group, the LFL group showed a markedly enriched pathway of starch and sucrose metabolism. The *bglX* gene was identified as a pivotal gene in this pathway, which regulated the conversion of cellobiose, cellodextrin, and β-D-glucoside into glucose. This metabolic process facilitated the accumulation of glucose, fructose, and starch. Enhanced starch accumulation facilitated the plant stress resistance by mitigating water stress in maize through thiourea application or salt tolerance by increasing starch accumulation in transgenic potatoes [54,55,56]. In this study, we observed a significant enrichment of differentially expressed genes related to the starch and sucrose metabolism pathway in LFL. The finding may indicate an adaptive response to the cooler climatic conditions of the LF region [57].

### 3.2. Transcriptional Variations Among Floral Organs from Distinct Regions Elucidate the Underlying Factors Contributing to Adaptive Defense

The comparative analysis of differential gene enrichment patterns between the HQO and LFO groups showed similarities to the enrichment patterns observed in the HQL/LFL group. The *bsa*A gene has been shown to play a crucial role in the glutathione metabolism pathway, where it modulates the interaction of glutathione with peroxides, thereby mitigating cellular damage induced by oxidative stress [58]. In this pathway process, glutathione disulfide is reduced back to glutathione through the action of NADPH, which decreases toxicity and enhances antioxidant capacity [59,60]. The validation of the *bsa*A gene in the HQO and LFO groups revealed that the corollas in the HQ region possessed more glutathione than those of the LF region. These findings suggest that the corolla of *R. decorum* from the HQ region possess significant antioxidant and detoxification properties, indicating their potential as candidates for the development of functional foods by the Bai nationality.

Within the phenylpropanoid biosynthesis pathway, the *CCR* gene plays a pivotal role in the biosynthesis of phenylpropanoids [61]. This biosynthetic pathway encompasses a wide variety of phenolic compounds and flavonoids, as well as their precursors, all of which are essential for plant defense mechanisms [62,63,64]. In the HQO group, the *CCR* gene exhibited significantly higher expression levels compared to the LFO group (Figure 4A), indicating that phenylpropanoids biosynthesis, which mediated plant defense mechanisms, was more active in the HQ region than in the LF region in response to minor environmental changes, despite minimal altitude differences.

Analogous to the HQL group, the *CYP75A* gene plays a crucial role in dihydroquercetin biosynthesis within the flavonoid biosynthesis pathway, thereby significantly influences the biosynthesis of downstream metabolites such as myricetin, (+)-gallocatechin, and (+)-catechin [37,38,39]. These flavonoids not only serve as essential defensive compounds for *R. decorum* during its flowering period, but also enhance its pharmaceutical value [65,66]. Moreover, *Rhododendron* species have been documented to exhibit potent insecticidal activities [67,68]. In this study, we observed that THE *CYP75A* gene exhibited significantly higher expression levels in the three floral organs in the HQ region. This indicated that *R. decorum* inhabiting this area might have greater defensive requirements compared to those in LF region.

Significant enrichment of differentially expressed genes was observed in the benzoxazinoid biosynthesis pathway within the HQO group. Notably, the *BX6* gene was identified as a pivotal regulator in this pathway. Additionally, benzoxazinoids are recognized as important secondary metabolites in herbaceous plants, functioning as natural pesticides [69]. In cereal crops such as maize and wheat, benzoxazinoids undergo further modifications mediated by the dioxygenase gene *BX6* in response to various pathogens and pests [70,71]. This defense mechanism involving benzoxazinoid biosynthesis in maize served to deter herbivores consumption [72] and exhibited a similar enrichment trend in the benzoxazinoid biosynthesis pathway in *R. williamsianum* [73]. The validation of the *BX6* gene demonstrated an expression pattern consistent with that of the *CYP75A* gene, showing significant expression in the HQ region. These results suggested that plants inhabiting the HQ area synthesized benzoxazinoid compounds as a defense against pests, potentially providing a valuable natural resource for pesticide development.

In the LFO group, a significant enrichment of differentially expressed genes was observed in three specific pathways: pentose and glucuronate interconversions, monoterpenoid biosynthesis, and starch and sucrose metabolism. Three genes, namely *USP*, *CYP76F14*, and *bglX*, were identified as pivotal components within these pathways. Specifically, the expression pattern of the *bglX* gene in the corolla of *R. decorum* was consistent with that observed in leaf samples from the LF region, indicating that both tissues exhibited a similar response to cold stress within the same region [57]. Furthermore, the pentose and glucuronate interconversions pathway exhibited significant enrichment of genes with functional variations relevant to this metabolic process. Notably, *USP*, a pivotal gene in this pathway, actively participates in glucose activation, thereby enhancing its efficient utilization [74]. Activated glucose serves as a substrate for the synthesis of polysaccharides, glycolipids, and glycoproteins in higher plants [75]. Consequently, the *USP* gene exhibited expression patterns similar to the *bglX* gene in both the pentose and glucuronate interconversions pathways and the starch and sucrose metabolism pathways, revealing the potential functional distinctions in corollas between the LF and HQ regions. These consistent findings indicate potential adaptations to the cool climatic conditions prevalent in this area.

Furthermore, a significant enrichment of differentially expressed genes was identified in the monoterpenoid biosynthesis pathway within the LFO group. Specifically, the *CYP76F14* gene plays a crucial role in the conversion of linalool to 8-hydroxylinalool and 8-oxolinalool in the corolla [76,77,78]. These aromatic compounds have been detected in the buds of *Camellia sinensis* var. *assamica* and the corolla of *Syringa vulgaris* [79,80]. Additionally, linalool and its derivatives are prominent aromatic components found in honey and wine [81,82], recognized for their anti-inflammatory and neuroprotective properties [83]. This highlights the potential value of *R. decorum* corolla as a source of functional foods or medicinal products. The *CYP76F14* gene demonstrated significantly elevated expression in the corolla of the LFO group, suggesting a substantial synthesis of linalool derivatives in high-altitude environments. Consequently, the transcriptional regulation of linalool biosynthesis in the corolla might represent an adaptive strategy to attract pollinating insects, which was critical for plant reproductive success and survival, especially in harsh environments where pollinator availability is limited [84,85].

## 4. Materials and Methods

### 4.1. Sample Collection and RNA Extraction

In May 2024, healthy materials of *R. decorum* were collected from Heqing County (HQ) (100.075720° E, 26.485620° N, Altitude 3038 m), Dali Bai Autonomous Prefecture and Yulong Naxi Autonomous County (LF) (100.180355° E, 27.001036° N, Altitude 3220 m), Lijiang City, Yunnan Province, China. Five healthy individuals of *R. decorum* were selected from each of the HQ and LF regions for sampling, respectively. For each individual, three to five intact flowers were collected and subsequently separated into the corolla samples (HQO and LFO) and the androecium/gynoecium samples (HQI and LFI). Additionally, three to five healthy fresh leaves (HQL and LFL) were also sampled from each individual plant. For each organ, to minimize organic difference within different plant, the leaves, corollas, and androecium/gynoecium from three to five matured individuals were collected, respectively. The same leaf samples from different individuals were pooled together, and then divided into three groups for sequencing, followed by being immediately flash-frozen in liquid nitrogen for preservation at −80 °C until use. The sampling strategy was employed to treat the corollas and androecium/gynoecium, respectively. The Qiagen RNeasy Plant Mini Kit manufactured by Qiagen company based in Hilden, Germany was utilized to isolate total RNA from approximately 5 g of each sample following the manufacturer’s instructions. The quantity and quality of extracted total RNA were assessed using a NanoDrop 2000 spectrophotometer (Thermo Fisher Scientific Co., Ltd., Waltham, MA, USA) and Agilent 2100 Bioanalyzer (Agilent Technologies, Santa Clara, CA, USA). RNA samples with a minimum RNA Integrity Number (RIN) value equal or greater than 8.0 along with a range between 1.8 and 2.0 for their respective absorbance ratios at wavelengths of both A260/A280 nm were chosen for subsequent cDNA library construction and sequencing [86].

### 4.2. cDNA Library Construction and Transcriptome Sequencing

The cDNA libraries were constructed using 1 μg total RNA from each sample, following the manufacturer’s recommendations for the NEBNext^®^Ultra™ RNA Library Prep Kit from Illumina^®^ (NEB, Ipswich, MA, USA). Initially, mRNA was purified from the total RNA and reverse transcribed into first-strand cDNA using random hexamer primers and M-MuLV Reverse Transcriptase in NEBNext First Strand Synthesis Reaction Buffer (5X). Subsequently, second-strand cDNA synthesis was performed with DNA Polymerase I and RNase H enzymes. Once the synthesized cDNA fragments reached an approximate length of 240 bp, they were further purified using the AMPure XP system (Beckman Coulter, Beverly, MA, USA). The blunt-ended cDNA fragments were then ligated with adapters containing a ‘T’ nucleotide overhang at their 3’ ends to generate paired-end libraries for sequencing. PCR amplification and enrichment of the library were carried out using adaptor region-based primers. The quality of the sequencing library was assessed on an Agilent Bioanalyzer 2100 system (Agilent Technologies, Santa Clara, CA, USA). Finally, the libraries were sequenced on an Illumina HiSeqXTM10 instrument (Illumina Inc., San Diego, CA, USA) at Biomarker Technologies Co., Ltd. (Beijing, China).

### 4.3. Transcriptome Assembly and Annotation

The clean reads were obtained by adapter trimming and removal of low-quality reads from the raw RNA sequencing data. Subsequently, de novo assembly was performed using Trinity software (version 2.14.0) to generate transcripts [87,88]. All transcripts underwent further analysis to eliminate redundancies and obtain non-redundant unigenes. These non-redundant unigenes were subjected to blast analysis against major public databases, including the NCBI non-redundant protein sequence database (NR database) (https://www.ncbi.nlm.nih.gov/protein/) (accessed on 1 July 2024) [89], Swiss Prot database (https://www.uniprot.org/uniprot/) (accessed on 1 July 2024) [90], Clusters of Orthologous Groups (COG) (http s://www.ncbi.nlm.nih.gov/research/cog) (accessed on 1 July 2024), Clusters of Protein homology (KOG) (https://ftp.ncbi.nlm.nih.gov/pub/COG/KOG/) (accessed on 1 July 2024) [91], eggNOG4 (http://eggnogdb.embl.de/) (accessed on 1 July 2024) [92], and Pfam (https://pfam.xfam.org) (accessed on 1 July 2024) [93], with a cut-off value below 10^−5^ for identification of homologous sequences. Gene Ontology categories were assigned using Blast2GO software (version 2.5) with default parameters [94]. Furthermore, TransDecoder software (v5.0.0) (https://transdecoder.github.io/) (accessed on 1 July 2024) was employed to predict coding region sequences (CDSs) based on alignment with protein domain sequences in the Pfam database for obtaining corresponding amino acid sequences.

### 4.4. Functional Annotation and Differential Expression Analysis

The assembled unigenes were functionally annotated by retrieving the entire identified unigenes from the Kyoto Encyclopedia of Genes and Genomes (KEGG) database [95]. A threshold E-value below 10^−5^ was set using BLASTX. Additionally, KOBAS software (version 3.0.3) was employed with default parameters to assign the unigenes into KEGG orthology (KO) for determining biosynthesis pathways. Gene expression levels were calculated using RNA-Seq by Expectation-Maximization (RSEM) software (version 1.3.3) to estimate fragments per kilobase per million fragments mapped (FPKM) values [96]. Differential expression analysis based on the negative binomial distribution was performed using DESeq2 software (version 1.30.1). The resulting *p*-values were adjusted using Benjamini and Hochberg’s approach to control false discovery rate, with a significance threshold of adjusted *p*-value < 0.05 assigned for identifying differentially expressed genes (DEGs) [97].

### 4.5. Validation of Pivotal Gene Expression by qRT-PCR

Validation of gene expression was conducted using quantitative real-time PCR (qRT-PCR). Total RNA from Different organs of *R. decorum* were extracted using the RNAprep Pure Plant Kit (Tiangen, Beijing, China). Reverse transcription was performed to generate cDNA with the HyperScriptTM III RT SuperMix for qPCR with gDNA Remover (NovaBio, Shanghai, China). For qRT-PCR experiments, the 2 × S6 Universal SYBR qPCR Mix (NovaBio, Shanghai, China) was utilized. This product is a premix containing enzymes, dNTPs, buffer solution, fluorescent dyes including SYBR Green I dye, and reference dyes along with other necessary components for qPCR. The final reaction volume of 20 μL consisted of 10 μL 2 × S6 Universal SYBR qPCR Mix, 2 μL first-strand cDNA template, 0.4 μL each of forward and reverse primers and 7.2 μL ddH_2_O. Actin served as an internal reference control for data normalization. The expression levels of target genes were determined using the 2^−ΔΔCt^ method [98]. All qRT-PCR experiments were performed in triplicate.

## 5. Conclusions

In this study, we analyzed the transcriptional differences in the floral organs (corolla and androecium/gynoecium) and leaves of *R. decorum* between two different regions. Despite the two study regions sharing similar climatic conditions, the gene expression patterns of *R. decorum* exhibited distinct trends during the flowering period. *Rhododendron decorum* in the HQ region exhibited an increased demand for plant immunity, which included resistance to diseases, insects, and herbivores across various plant organs. Conversely, in the LF region, they showed a greater sensitivity to the environmental factors, such as cold tolerance, allelochemical production, and attraction to pollinators. The functional concordance of differentially expressed genes across the various organs aligns with the observed distribution pattern of *R. decorum*. The differential expression of transcriptome genes elucidated the underlying factors contributing to regional variations in adaptive defense mechanism of *R. decorum*. These findings provided valuable insights into further utilization of *R. decorum* resources.

## Figures and Tables

**Table 1:** Annotation of identified unigenes against eight different databases.

Annotated Database	Annotated Number	Percentage of Annotated Genes (%)
COG	8369	9.09
GO	27,221	29.58
KEGG	23,369	25.39
KOG	19,916	21.64
Pfam	25,018	27.19
Swissprot	23,521	25.56
TrEMBL	40,563	44.08
eggNOG	30,146	32.76
nr	40,574	44.09
All Annotated	43,515	47.29

## Data Availability

The data presented in this study are available on request from the corresponding author. The data are not publicly available due to privacy.

## Supplementary Materials

The following supporting information can be downloaded at: https://www.mdpi.com/article/10.3390/plants14040559/s1, Figure S1: The length distribution of identified unigenes and CDS sequences; Figure S2: The statistical analysis of differentially expressed genes between different groups; Figure S3: The KEGG classification of differentially expressed genes within different groups; Table S1: Statistical evaluation for sample sequencing data; Table S2: The results of transcriptome sequencing data assembly; Table S3: The length distribution of identified CDS sequences; Table S4: The FPKM value estimation of identified DEGs within different groups; Table S5: The KEGG enrichment analysis of differentially expressed genes within group comparison.

## Abbreviations

The following abbreviations are used in this manuscript:

HQ	Heqing County, Dali Bai Autonomous Prefecture, Yunnan Province
LF	Yulong Naxi Autonomous County, Lijiang City, Yunnan Province
CDSs	Coding sequences
COG	The database of Clusters of Orthologous Groups of proteins
GO	Gene Ontology database
KEGG	The database of Kyoto Encyclopedia of Genes and Genomes
KOG	The database of Clusters of Protein homology
Pfam	The database of Homologous protein family
Swissprot	A manually annotated, non-redundant protein sequence database
TrEMBL	Translation of EMBL Nucleotide Sequence Database
eggNOG	A database of orthologous groups of genes
nr	Non-redundant protein sequence database
FPKM	Fragments Per Kilobase of exon model per Million mapped fragments
PCA	Principal component analysis
HQI	Androecium/gynoecium group in Heqing County
LFI	Androecium/gynoecium group in Yulong Naxi Autonomous County
HQL	Leaf group in Heqing County
LFL	Leaf group in Yulong Naxi Autonomous County
HQO	Corolla group in Heqing County
LFO	Corolla group in Yulong Naxi Autonomous County
DEGs	Differentially expressed genes
MAPK	Mitogen-activated protein kinase
SNARE	Soluble N-ethylmaleimide-sensitive factor attachment protein receptor
CO	A circadian clock-regulated gene encoding a transcription factor
NADPH	Nicotinamide adenine dinucleotide phosphate
