# Supplementary figures and images for: Adaptive Defense Mechanism During Flowering Period of Rhododendron decorum Revealed by Comparative Transcriptomic Analysis
Source: Plants (Basel). 2025 Feb 12;14(4):559. doi: 10.3390/plants14040559 (PMC11859546; doi:10.3390/plants14040559)

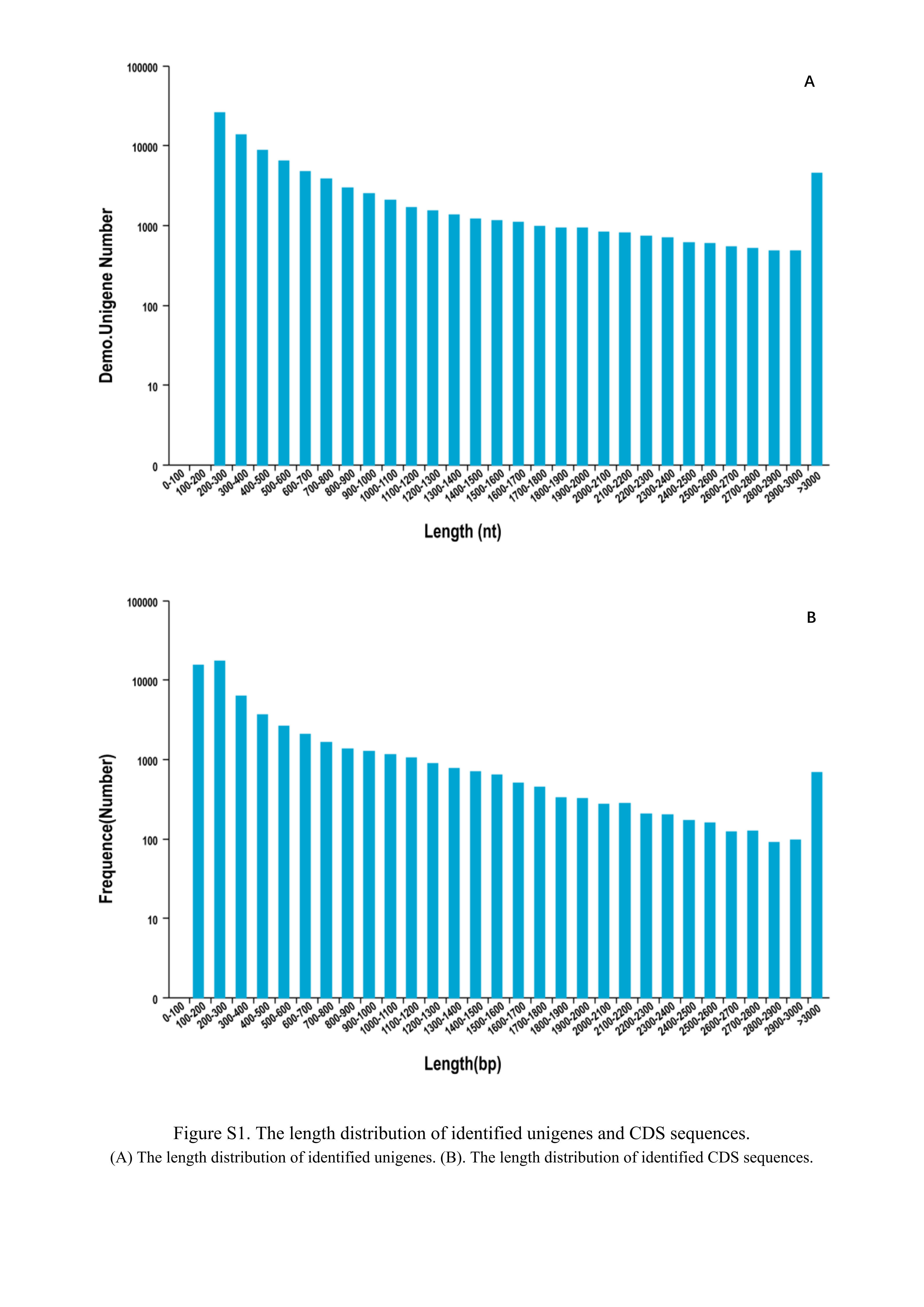

Supplement: Supplementary file 1 [file plants-14-00559-s001.zip › Figure S1.jpg]

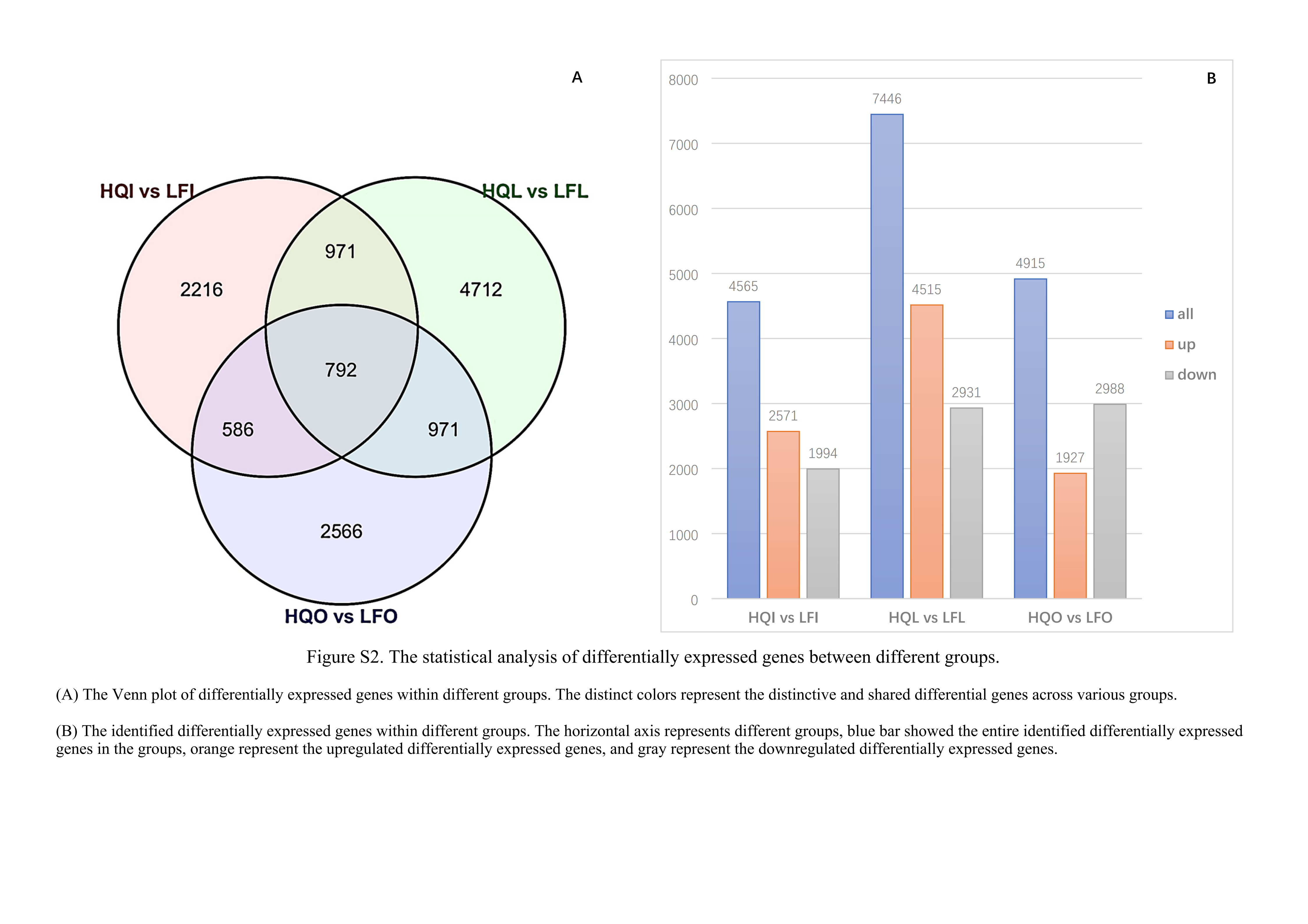

Supplement: Supplementary file 1 [file plants-14-00559-s001.zip › Figure S2.jpg]

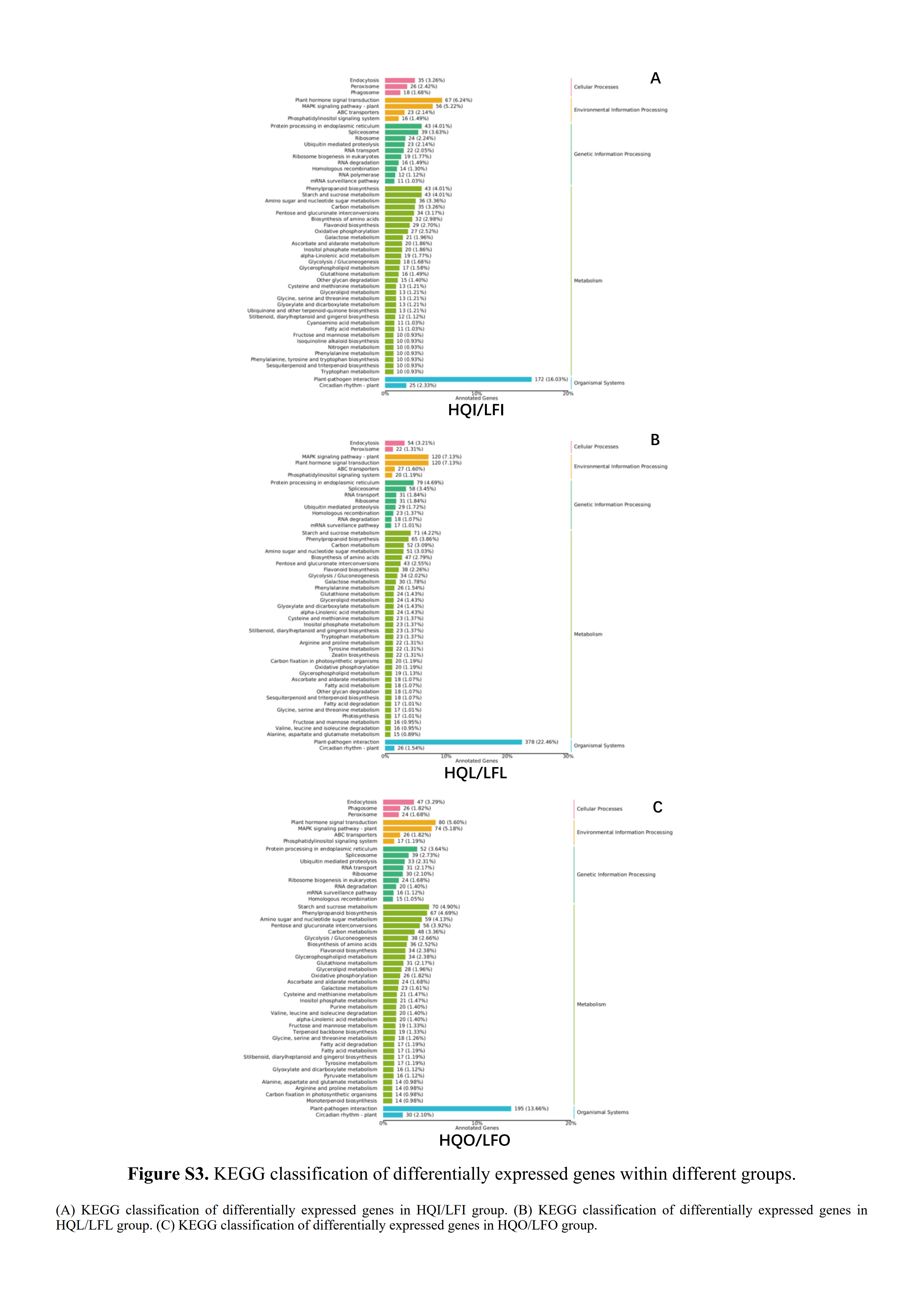

Supplement: Supplementary file 1 [file plants-14-00559-s001.zip › Figure S3.jpg]
